# Radiation of members of the *Soroserishookeriana* complex (Asteraceae) on the Qinghai-Tibetan Plateau and their proposed taxonomic treatment

**DOI:** 10.3897/phytokeys.114.29914

**Published:** 2018-12-20

**Authors:** La-Mei Heng, Yu-Lin Zheng, Yong-Bao Zhao, Yu-Jin Wang

**Affiliations:** 1 State Key Laboratory of Grassland Agro-Ecosystem, School of Life Sciences, Lanzhou University, Lanzhou 730000, PR China Lanzhou University Lanzhou China

**Keywords:** Radiation, *Soroserishookeriana* complex, taxonomic treatment, Asteraceae

## Abstract

The existence of intermediate types is a major obstacle that can hinder the circumscription of species. Elucidating the mechanism responsible for intermediate types is essential for achieving a reasonable taxonomical treatment. In this study, we explored the evolutionary history and taxonomic treatment of the *Soroserishookeriana* (C.B.Clarke) Stebbins complex, which comprises six named taxa that may be taxonomically distinct and are all native to the Qingha-Tibetan Plateau (QTP). We made an investigation across the distribution range of *Soroseris* Stebbins and sampled 27 populations, mostly from the complex. Internal transcribed spacer (ITS) and two chloroplast loci were sequenced and analysed using the neighbour-joining and Bayesian inference methods. The resulting phylogenies show no well supported inconsistence in topologies, in line with the lack of incongruence detected by the length difference test. However, all the trees were largely unresolved within *S.hookeriana* complex, irrespective of the optimality criterion employed. We interpret these results as an experience of radiation, which is a common process for native genera on the QTP. Thus, we suggest that all of the morphotypes might be different forms, generated by incipient speciation due to recent explosive differentiation, possibly triggered by the drastic environmental changes of the QTP. Given their evolutionary history, we propose a pragmatic method for treating all of these species as subspecies with a total of four new combinations.

## Introduction

The description and delimitation of species in an evolutionary framework is essential for understanding patterns of biodiversity and distribution, as well as when assessing conservation strategies for natural resources (Liu 2016; Yang 2016). However, species complexes, comprising a few distinct morphotypes with a series of intermediates at the species level, are a difficult problem for taxonomists (Liu 2016). These intermediates might be derived via various mechanisms such as intraspecies variation, interspecies hybridisation, convergent evolution or radiation (Wang et al. 2004; Liu et al. 2006). Increasing studies suggest that DNA sequences can be employed to elucidate the mechanisms responsible for intermediate types (Su et al. 2015; Zheng et al. 2017).

*Soroseris* is a genus comprising seven species and all are endemic to the Qingha-Tibetan Plateau (QTP) according to the latest comprehensive revision (Shi and Kilian 2011). Despite its restricted distribution and the paucity of species, this genus contains two species complexes. The first referred to as the *Soroserisglomerata* (Decne.) Stebbins complex comprises *S.glomerata* and five possibly distinct species, all of which have been treated as *S.glomerata* in some studies (Stebbins 1940; Shih 1997; Shi and Kilian 2011). Two were recognised as independent species in the latest revision, i.e. *S.pumila* Stebbins and *S.depressa* (Hook. f. & Thomson) J. W. Zhang, N. Kilian & H. Sun, whereas three, i.e. *S.bellidifolia* (Hand.-Mazz.) Stebbins, *S.deasyi* Stebbins, and *S.rosularis* (Diels) Stebbins, were accepted as synonyms with a comment that it is appropriate to recognise them as subspecies, awaiting more studies on variation and distribution (Shi and Kilian 2011). Phylogenetic studies, based on either nuclear the internal transcribed spacer (ITS) or plastid regions, showed that *S.glomerata* could be resolved into at least two distantly related clades (Zhang et al. 2011), thereby implying that it may not be monophyletic.

The second species complex, referred to as the *S.hookeriana* (C.B.Clarke) Stebbins complex, comprises *S.hookeriana* and five possibly independent species, where one was accepted as *S.erysimoides* (Hand.-Mazz.) C. Shih in the latest revision, whereas the other four, i.e. *S.occidentalis* (Stebbins) Tzvelev, *S.hirsuta* (J. Anthony) C. Shih, *S.gillii* (S. Moore) Stebbins and S.gilliisubsp.handelii Stebbins, were treated as synonyms of *S.hookeriana* (Shi and Kilian 2011). Several other treatments have been proposed and we listed four of them in Table 1 (Stebbins 1940; Shih 1993, 1997; Shi and Kilian 2011). These taxa have all been treated at species rank except for S.gilliisubsp.handelii, yet in other treatments have been treated as synonyms or subspecies, notably, under different species (Stebbins 1940; Shih 1993; Tzvelev 2007; Shi and Kilian 2011). This complicated taxonomical controversy undoubtedly reflects the difficulty in delimitating taxa within *Soroseris* in terms of their morphology.

**Table 1.** Different taxonomical treatments of the possible members of the *Soroserishookeriana* complex. FRPS: Flora Reipublicae Popularis Sinicae; FOC: Flora of China.

Stebbins (1940)	Shih C (1993)	FRPS (1997)	FOC (2011)
S. gillii subsp. typica	* S. trichocarpa *	* S. gillii *	* S. hookeriana *
S. gillii subsp. occidentalis	* S. hirsuta *	* S. hirsuta *	* S. hookeriana *
S. gillii subsp. hirsuta	* S. hirsuta *	* S. hirsuta *	* S. hookeriana *
S. gillii subsp. handelii	* S. hirsuta *	* S. hirsuta *	* S. hookeriana *
S. hookeriana subsp. typica	* S. hookeriana *	* S. hookeriana *	* S. hookeriana *
S. hookeriana subsp. erysimoides	* S. erysimoides *	* S. erysimoides *	* S. erysimoides *
* S. bellidifolia *	* S. hirsuta *	* S. hirsuta *	* S. glomerata *

In addition to the controversial treatments mentioned above, the circumscription of *Soroseris* is also disputed. For example, two species of *Syncalathium* Lipschitz are included in *Soroseris* in some systems (Shih 1993). Recently, a number of studies based on pollen, achene morphology, karyotypes and multiple DNA loci (Zhang et al. 2007, 2013; Zhang and Sun 2011; Peng et al. 2013) have supported the circumscription of the latest revision of *Soroseris* (Shi and Kilian 2011), but there are some slight differences compared with the first revision (Stebbins1940). In morphological terms, the genus is circumscribed mainly based on a densely crowded capitula on a thick and hollow stem, with two layers of phyllaries, where the outer layer are much smaller (Stebbins 1940). Molecular phylogenetic analyses indicate that *Syncalathium* might be the sister group of *Soroseris*, in line with their similarity in morphology, such as densely crowded capitula (Zhang et al. 2011), the chromosome number and the preferred habitat in high altitude (Zhang et al. 2007; Ying and Yang 2011; Yang et al. 2017).

Previous studies have resolved the circumscription and sister (*Syncalathium*) of *Soroseris*, but the delimitation within the two species complexes remains unresolved (Zhang and Sun 2011). A major problem is the lack of samples of multiple individuals and comparisons of intra-/interspecies genetic diversity (Zhang and Sun 2011). In this study, we focused on the *S.hookeriana* complex. We sampled multiple individuals and sequenced several loci in order: (1) to clarify the mechanisms responsible for the complicated relationships in terms of morphology in this species complex; and (2) to revise the taxonomy of the *S.hookeriana* complex. We supposed that, if hybridisation was documented, the parental species and the possible cases of hybridisation could be recognised or, if radiation was indicated, the number of species within the complex could be greatly reduced.

## Materials and methods

### Taxon sampling

In total, from the QTP, we collected 35 individuals from 27 populations belonging to *Soroseris* and two individuals from *Syncalathium* as an outgroup, according to a previous study (Zhang et al. 2011) and all the voucher specimens were deposited in the herbarium of Lanzhou University. The samples from *Soroseris* were identified as belonging to six species, with five from the latest revision (Shi and Kilian 2011) and one that differed from all the known species (Voucher: CY40). The members of *S.hookeriana* complex, *S.hookeriana* and *S.erysimoides*, total up to thirty individuals and they could be further sorted into at least eight morphotypes. Six of them are largely comparable to six subspecies recognised by Stebbins (Stebbins 1940), although more or less variations exist. Two of them seem to intermediate amongst different subspecies and here we named them Intermediate A and B, tentatively. A morphological comparison amongst these specimens, together with several related ones, is listed in Suppl. material 1. In addition, sequences from 17 individuals belonging to three species, including nine from the *S.hookeriana* complex and one we failed to collect, i.e. *S.umbrella* (Franch.) Stebbins, were downloaded from GenBank, which were all obtained in the study by Zhang et al. (Zhang et al. 2011). All of the samples, voucher locations and GenBank numbers used in the analysis are listed in Table 2.

### DNA extraction, PCR amplification, and sequencing

Genomic DNA was extracted from dried leaves in silica gel using the CTAB method (Doyle and Dickson 1987). Three regions (*psbA-trnH*, *matK* and ITS) were amplified and sequenced with the primers from published literature (White et al. 1990; Sang et al. 1997; Ford et al. 2009). The PCR reaction mixture comprising 25 μl was prepared and amplified according to the procedure described by Wang et al. (Wang et al. 2009). The PCR products were sent to the Beijing Genomics Institute for commercial sequencing. Sequences were aligned using CLUSTALX v.2.1 (Thompson et al. 1997) with the default settings and adjusted manually with Bioedit v.7.0.5 (Hall 1999). All of the sequences were registered in GenBank (Table 2).

**Table 2.** Taxa, collection localities, vouchers (or the references for those downloaded from NCBI) and their GenBank accession numbers.

Taxon (FOC, 2011)	Collection locality	Latitude (°N) / Longitude (°E)	Altitude (m)	Voucher	Genbank number (ITS, *matK*, *psbA-trnH*)
* S. erysimoides *	Cuona, Tibet, China	27.9269, 91.8789	4519	Y.-J. Wang, CN30 (LZU)	MG932859; MG946722; MG932893
	Cuona, Tibet, China	27.9269, 91.8789	4519	Y.-J. Wang, CN47 (LZU)	MG932861; MG946724; MG932895
	Cuona, Tibet, China	27.9269, 91.8789	4519	Y.-J. Wang, CN48 (LZU)	MG932862; MG946725; MG932896
	Cuona, Tibet, China	27.9269, 91.8789	4519	Y.-J. Wang, CN49 (LZU)	MG932863; MG946726; MG932897
	Cuona, Tibet, China	27.9269, 91.8789	4519	Y.-J. Wang, CN50 (LZU)	MG932864; MG946727; MG932898
	Cuona, Tibet, China	27.9269, 91.8789	4519	Y.-J. Wang, CN51 (LZU)	MG932865; MG946728; MG932899
	Geermu, Qinghai, China	35.4158, 96.3409	4665	Y.-J. Wang, GEM3 (LZU)	MG932858; MG946721; MG932892
	Yadong, Tibet, China	27.5518, 88.9306	3059	Y.-J. Wang, YD46 (LZU)	MG932860; MG946723; MG932894
	Xingu, Sichuan, China	–	–	Zhang et al. 2011	HQ436213; JF956518; HQ436180
	Tibet, China	–	–	Zhang et al. 2011	JF978800; JF956516; JN047244
	Deqin, Yunnan, China	–	–	Zhang et al. 2011	HQ436212; JF956517; HQ436179
	Sichuan, China	–	–	Zhang et al. 2011	JF978799; JF956515; JN047243
* S. hookeriana *	Chayu, Tibet, China	29.3252, 97.0390	4705	Y.-J. Wang, CY39 (LZU)	MG932868; MG946742; MG932910
	Chayu, Tibet, China	29.3252, 97.0390	4705	Y.-J. Wang, CY53 (LZU)	MG932869; MG946743; MG932917
	Daocheng, Sichuan, China	29.2953, 100.1466	4404	Y.-J. Wang, DC9 (LZU)	MG932871; MG932921; MG932921
	Kangding, Sichuan, China	29.4446, 101.4339	4657	Y.-J. Wang, KD11 (LZU)	--; MG946729; MG932900
	Kangding, Sichuan, China	30.0411, 101.9532	2861	J.-Q. Liu, KD54 (LZU)	MG932870; MG946732; MG932918
	Kangding, Sichuan, China	30.0411, 101.9532	2861	Y.-J. Wang, KD7 (LZU)	MG932877; MG946750; MG932914
	Xiangcheng, Sichuan, China	28.9312, 99.7835	2927	Y.-J. Wang, XC10 (LZU)	MG932876; MG946747; MG932915
	Xiaojin, Sichuan, China	30.5473, 102.5373	4519	Y.-J. Wang, XJ4 (LZU)	MG932873; MG946739; MG932911
	Xiaojin, Sichuan, China	30.5473, 102.5373	4519	Y.-J. Wang, XJ5 (LZU)	MG932874; MG946740; MG932914
	Xiaojin, Sichuan, China	30.5473, 102.5373	4519	Y.-J. Wang, XJ6 (LZU)	MG932875; MG946741; MG932920
	Zhiduo, Qinghai, China	33.5845, 96.3409	4689	Y.-J. Wang, ZD2 (LZU)	MG932866; MG932902; MG932902
	Sichuan, China	–	–	Zhang et al. 2011	HQ446097; JF956522; JN047246
	Sichuan, China	–	–	Zhang et al. 2011	HQ436227; JF956521; JN047245
	Kangding, Sichuan, China	–	–	Zhang et al. 2011	HQ436214; JF956520; HQ436181
	Cuomei, Tibet, China	28.7853, 91.7549	5048	Y.-J. Wang, CN25 (LZU)	MG932883; MG946734; MG932905
	Dingri, Tibet, China	28.5755, 87.1136	4305	Y.-J. Wang, DR55 (LZU)	MG932886; MG946737; MG932919
* S. hookeriana *	Dangxiong, Tibet, China	29.9018, 90.1370	5400	Y.-J. Wang, DX17 (LZU)	MG932882; MG946733; MG932901
	Dangxiong, Tibet, China	29.9018, 90.1370	5400	Y.-J. Wang, DX43 (LZU)	MG932885; MG946736; MG932912
	Longzi, Tibet, China	28.6027, 92.2142	4906	Y.-J. Wang, LZ27 (LZU)	MG932884; MG946735; MG932906
	Longzi, Tibet, China	28.6371, 92.2175	5106	Y.-J. Wang, LZ52 (LZU)	MG932878; MG946749; MG932916
	Yadong, Tibet, China	27.5527, 88.9315	3059	Y.-J. Wang, YD21 (LZU)	MG932867; MG946731; MG932904
	Hongshan, Yunnan, China	–	–	Zhang et al. 2011	HQ436218; JF956532; HQ436185
	Tibet, China	–	–	Zhang et al. 2011)	JF978806; JF956530; JN047250
	Longzi, Tibet, China	28.6371, 92.2175	5106	Y.-J. Wang, LZ33 (LZU)	MG932872; MG946738; MG932909
	Cuona, Tibet, China	27.9269, 91.8788	4519	Y.-J. Wang, CN29 (LZU)	MG932880; MG946745; MG932907
	Cuona, Tibet, China	27.8476, 91.8929	4732	Y.-J. Wang, CN32 (LZU)	MG932881; MG946746; MG932908
* S. glomerata *	Angren, Tibet, China	29.5021, 86.2770	4753	Y.-J. Wang, AR18 (LZU)	MG932887; MG946744; MG932922
	Tibet, China	–	–	Zhang et al. 2011	JF978802; JF956523; JN047247
	Daxueshan, Yunnan, China	–	–	Zhang et al. 2011	HQ436217; JF956527; HQ436184
	Deqin, Yunnan, China	–	–	Zhang et al. 2011	HQ436216; JF956528; HQ436183
	Tibet, China	–	–	Zhang et al. 2011	JF978804; JF956525; JN047248
* S. teres *	Yadong, Tibet, China	27.5503, 88.9316	3059	Y.-J. Wang, YD44 (LZU)	MG932888; MG946752; MG932924
	Yadong, Tibet, China	27.5503, 88.9316	3059	Y.-J. Wang, YD45 (LZU)	MG932889; MG946753; MG932925
* S. umbrella *	Zhonggashan, Yunnan, China	–	–	Zhang et al. 2011	HQ436197; HQ436164; HQ436131
	Hongshan, Yunnan, China	–	–	Zhang et al. 2011	HQ436198; HQ436165; HQ436132
*Soroseris* sp.	Chayu, Tibet, China	29.3252, 97.0390	4705	Y.-J. Wang, CY40 (LZU)	MG932879; MG946748; MG932923
* Syncalathium disciforme *	Heishui, Sichuan, China	32.1326, 102.3633	4016	Y.-J. Wang, HS12 (LZU)	MG932890; MG946754; MG932926
* Syncalathium kawaguchii *	Luozha, Tibet, China	28.2504, 91.0481	4112	Y.-J. Wang, LZ24 (LZU)	MG932891; MG946755; MG932927

### Data analysis

Three datasets were constructed for the ITS sequences, the combination of *psbA–trnH* and *matK* and the combination of all the three fragments. For the first two datasets, genetic distance was calculated with Mega (Tamura et al. 2013) under Kimura’s two-parameter (K-2P) model (Kimura 1980). For the last one, the congruence between ITS and the other two fragments was evaluated using the incongruence length difference (ILD) test in PAUP* 4.0b10. For all the three data sets, neighbour-joining (NJ) and Bayesian inference (BI) methods were employed to reconstruct the phylogenetic relationships. The NJ trees were built using PAUP version 4b10 with K-2P model (Swofford 2003). Node support was assessed based on bootstrap percentages (BP) of 100000 replicates. BI was implemented using MrBayes on XSEDE (v3.2.6) (Ronquist et al. 2012) and the optimal models for each marker were determined according to Akaike’s information criterion (Akaike 1974) using jModelTest2 on XSEDE (v2.1.6) (Darriba et al. 2012).

## Results

### ITS sequences

**Figure 1.**
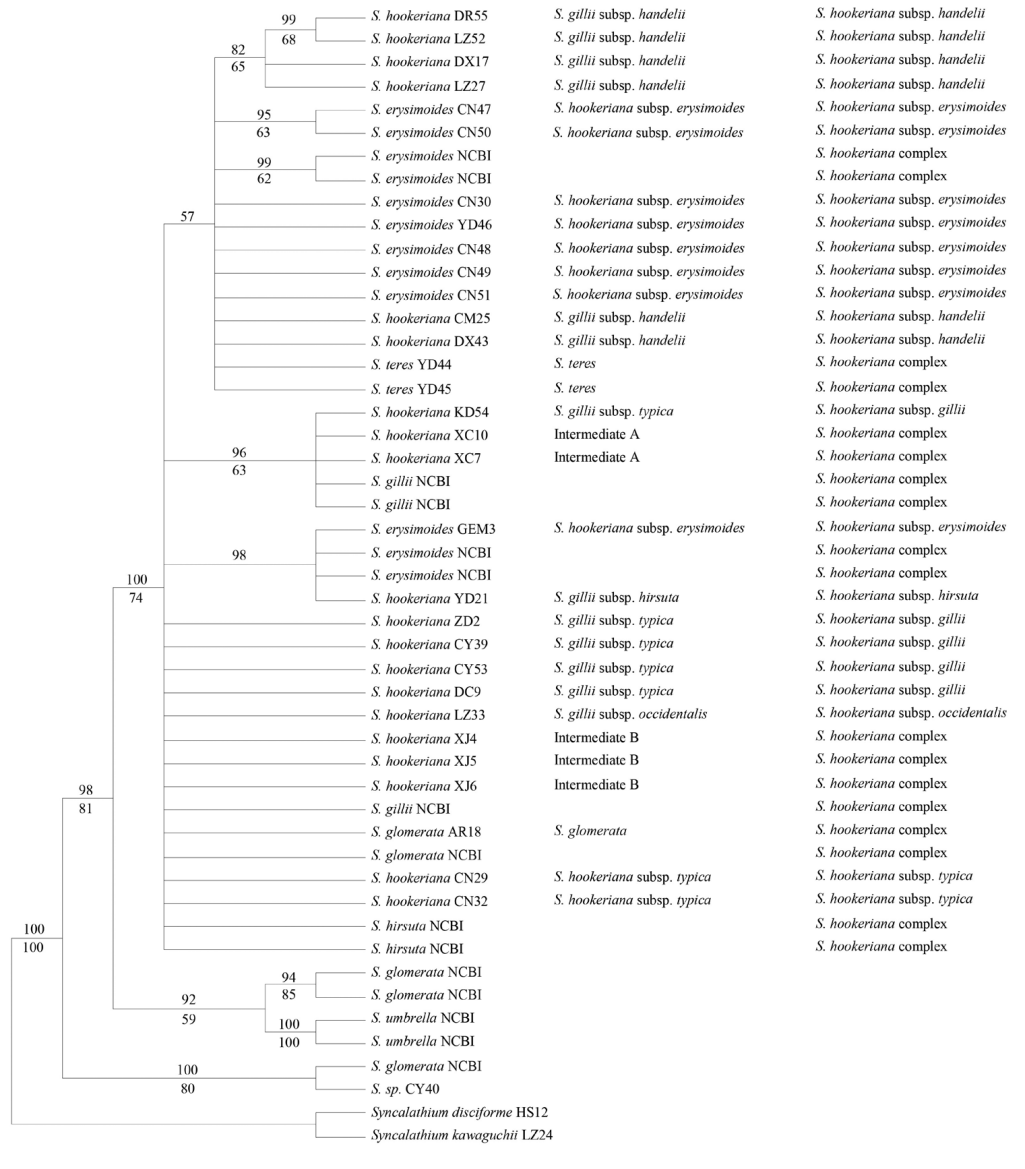
The 50% majority rule consensus tree derived from Bayesian inference of the nuclear internal transcribed spacer. Posterior probabilities and bootstrap percentages are indicated above and below the branches, respectively. The samples named according to FOC (2011) or NCBI, Stebbins (1940) and the present study are listed from left to right.

The aligned ITS dataset comprised 607 base pairs (bp) with 58 variable sites, where 36 sites were potentially parsimony informative. A total of 12 mosaic sites are detected from eight individuals, mostly with one or two. The K-2P distance, ranged from 0 to 2.4%, is 0.6% on average within the ingroup, while 0.3% on average or 1% maximally within the complex. The NJ tree was mostly congruent in terms of its topology with the 50% majority rule consensus tree derived from Bayesian analysis and the latter is shown in Fig. 1. The in-group samples were resolved into three clades. The first clade, which was a sister to the other two, comprised part of *S.glomerata* and a species that we failed to identify (BI = 100%, BP = 80%). The second clade contained two *S.glomerata* and *S.umbrella* sequences (BI = 92%, BP = 59%). The third clade comprised all of the others, including two *S.glomerata* individuals, two *S.teres* C. Shih individuals and all 37 from the *S.hookeriana* complex (BI = 100%, BP = 74%) but there was little resolution within this clade. Excluding *S.umbrella*, none of the species with multiple individual samples was recovered as monophyletic and *S.glomerata* samples were present in all three clades.

### Combined psbA–trnH and matK sequences

**Figure 2.**
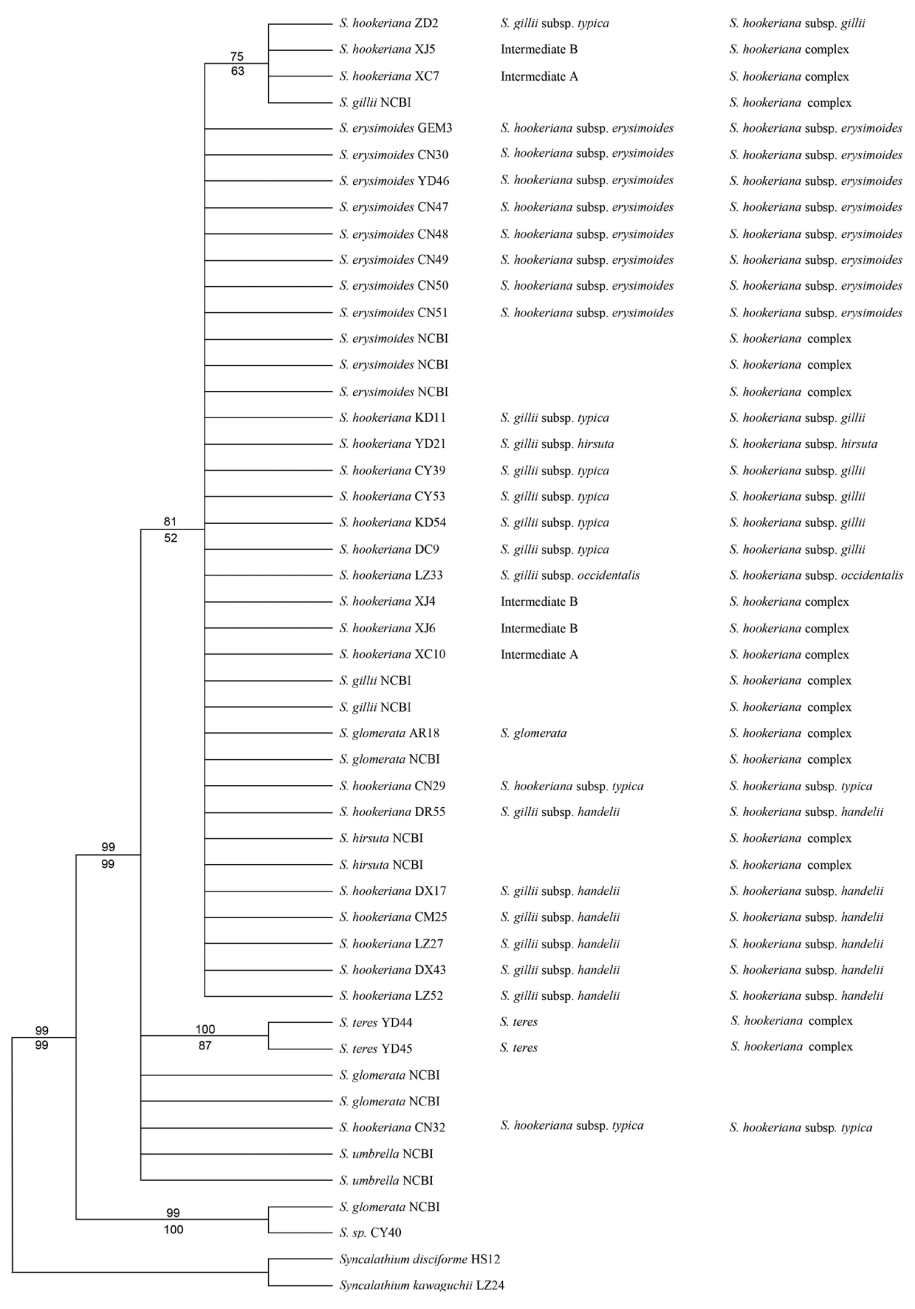
The 50% majority rule consensus tree derived from Bayesian inference of the combined sequences of *psbA-trnH* and *matK*. Posterior probabilities and bootstrap percentages are indicated above and below the branches, respectively. The samples named according to FOC (2011) or NCBI, Stebbins (1940) and the present study are listed from left to right.

The combined *psbA–trnH* and *matK* sequences measured 870 bp, where 54 nucleotide sites were variable and 23 were phylogenetically informative. The K-2P distance is estimated to be 0.2% on average and ranged from 0 to 1.8% within the ingroup, while 0.1% on average or 0.6% maximally within the complex. The NJ tree was congruent with the 50% major consensus tree obtained by BI and the latter is presented in Fig. 2. The topology recovered was very similar to that for ITS on the phylogenetic context of *S.hookeriana* complex, but two, one containing *S.umbrella* and the one containing *S.hookeriana* complex, of the three clades based on the ITS sequences, were combined as one.

### Combined ITS, psbA-trnH and matK sequences

ILD test (P = 0.289000) detected no strong evidence of incongruence between the data partitions. Thus, the three fragments are combined and the resulting topologies from NJ and BI (Suppl. material 2) are concordant. Being highly similar to that from ITS, three major clades within *Soroseris* were recovered and the relationship within *S.hookeriana* complex remains largely unresolved.

## Discussion

Aside from *S.umbrella*, no species were recovered in a monophyletic clade. In particular, *S.glomerata* was revealed to be present in all the three major clades (Figs 1, 2, Suppl. material 2), indicating that the circumscription of this species needed to be revised. All the members of the *S.hookeriana* complex formed a clade together with *S.teres* and part of *S.glomerata* (Figs 1, 2, Suppl. material 2), without subclades corresponded to the circumscription in the Flora of China or any other systems (Stebbins 1940; Shih 1993). Thus, the complex needs to be expanded to include all these members. However, the poor resolution was not sufficient to aid our selection from the proposed systems, although our results are helpful for understanding the processes or mechanisms responsible for the high variation within the complex.

In most cases, hybridisation is considered to explain the occurrence of intermediates. It is not possible to exclude this mechanism in the *S.hookeriana* complex, but it appears to conflict with the status of *Soroseris* because of the following reasons. First, hybridisation often results in different topologies when phylogenetic trees are reconstructed based on ITS and chloroplast sequences, which was not the case for *Soroseris*. Second, hybridisation might only affect the tree obtained based on a nuclear marker, but the grouping of the chloroplast sequences was also not species-specific for *Soroseris*. Third, the occurrence of hybridisation might be determined by the distribution of the parent species, where it usually occurs in areas where the ranges of the two species meet and thus the diversity of these populations might be higher than that of others. We found no evidence of hybridisation based on these three reasons in *Soroseris*. In addition, mosaic sites in nuclear ITS sequences, which are characteristic of many taxa generated by hybridisation, are rare in *Soroseris*.

Alternatively, we suggest that radiation might be the main mechanism responsible for the various forms of intermediates in *Soroseris*. Radiation involves the rapid differentiation of a lineage within a short time interval, which is mostly triggered by environmental change or morphological innovation (Liu et al. 2006). The rapid uplift of the QTP generated a large number of heterogeneous environments and promoted the rapid differentiation of genera such as *Rhododendron* L. (Milne 2004), *Ligularia* Cass (Liu et al. 2006) and *Saussurea* DC (Wang et al. 2009). The main typical characteristic of these genera is a poorly resolved phylogeny with a large number of parallel branches, as well as complicated but subtle morphological variation amongst populations or species (Wang et al. 2009). However, only a few variations might exist within a population or certain region, whereas hybridisation is characterised by high variation within a population (Meeus et al. 2016). In the present study, few morphological variations were detected in each *Soroseris* population, whereas many were found between populations, particularly in terms of the leaf shape, indumentum in the phyllary and the plant height. Two or more states were present for all three of these characters and various combinations were present in different populations. We consider that all the populations of the *S.hookeriana* complex might have been derived from the same widespread ancestor on the QTP, but various environmental changes following the uplift of the QTP reduced the gene flow amongst most of the populations to yield a number of parallel branches, while adaptation to the local environment also resulted in an array of morphotypes, which were treated as subspecies, possibly under different species, by different systems (Stebbins 1940; Shih 1997; Shi and Kilian 2011).

According to the phylogenetic context and little genetic differentiation (ITS: 0.3% on average while 1% maximally; concatenated cp: 0.1% on average while 0.6% maximally), all members of the *S.hookeriana* complex (include *S.teres* and part of *S.glomerate*) could be treated as single species. However, this revision will make it difficult to describe an assemblage. In addition, this treatment might fail to reflect the evolutionary history discussed above and the biodiversity may be underestimated. However, the alternative treatment is also not perfect because separating all of the species will make identification difficult, especially when encountering intermediates, which is common in the field. In order to address these issues, we propose to treat all of the morphotypes, especially those with the typical morphology and widespread distribution, as subspecies of *S.hookeriana* because this is the earliest name of a species reported within the complex. However, we abandoned, for the time being, assigning new names to *S.teres* and *S.glomerate* due to insufficient sampling as well as distinct morphology. In addition, the name S.hookerianasubsp.erysimoides (Hand.-Mazz.) Stebbins has been published previously and we suggest that it is restored. Thus, a total of eight taxa, including four new combinations, are proposed and a key is provided in the following.

### Key to the possible members of the *S.hookeriana* complex

**Table:** 

1a	Cataphylls numerous on the lower part of the stem; leaf blades elliptic or spatulate; ligule of corollas mostly equal to or shorter than the tube	***S.glomerata* (only those closely related to the *S.hookeriana* complex)**
1b	Cataphylls few or none; leaf blades lanceolate or oblanceolate; ligules distinctly exceeding the tube of the corolla	**2**
2a	Synflorescence elongate and cylindric	*** S. teres ***
2b	Synflorescence hemispheric	**3**
3a	Leaves entire or denticulate, obtuse at the apex; upper leaves, bracts of the inflorescence and peduncles glabrous or sparingly hirsute	** S. hookeriana subsp. erysimoides **
3b	Leaves pinnatifid, acute at the apex; upper leaves, bracts of the inflorescence and peduncles strongly hirsute4a. Involucral bracts sparsely to strongly hirsute	**4**
4a	Involucral bracts sparsely to strongly hirsute	**5**
5a	Leaves sinuate-pinnatifid, sinuate-dentate or merely denticulate; inner bracts sparsely to moderately hirsute	**S.hookerianasubsp.occidentalis (new combination)**
5b	Leaves runcinate-pinnatifid; inner bracts densely hirsute	**6**
6a	Stem tall, 4–20 cm; leaf blade pinnately lobed, lobes narrowly triangular	** S. hookeriana subsp. typica **
6b	Stem short, less than 6 cm tall; leaf blade pinnately lobed, lobes irregular	**S.hookerianasubsp.hirsuta (new combination)**
4b	Involucral bracts glabrous	**7**
7a	Leaf blade 3–8cm long, 0.7–1.8 cm wide	**S.hookerianasubsp.gillii (new combination)**
7b	Leaf blade 2–4cm long, 0.5–1.3 cm wide	**S.hookerianasubsp.handelii (new combination)**

#### Soroseris hookeriana subsp. gillii

Taxon classificationPlantaeAsteralesAsteraceae

(S.Moore) Yu.J. Wang & L.M. Heng, comb. et stat. nov.

urn:lsid:ipni.org:names:60477690-2

≡Crepisgillii S. Moore in Journ. Bot. 37: 170. 1899 (Syntype: K000250191); ≡Soroserisgillii (S. Moore) Stebbins in Mem. Torrey Bot. Club 19 (3): 41. 1940; S. Y. Hu in Quart. Journ. Taiwan Mus. 21 (3–4): 166. 1968; Higher Plants of China 4: 686, figure 6786. 1975; Flora Reipublicae Popularis Sinicae. 80 (1): 199. 1997; ≡Soroserisgillii(S. Moore)Stebbinssubsp.typica Stebbins in Mem. Torrey Bot. Club. 19 (3): 42. 1940; S. Y. Hu in Quart. Journ. Taiwan Mus. 21 (3–4): 166. 1968; ≡Soroseristrichocarpa (Franch.) Shih in Act. Phytotax. Sin 31: 446. 1993; Flora Reipublicae Popularis Sinicae. 80 (1): 199. 1997.

#### Soroseris hookeriana subsp. hirsuta

Taxon classificationPlantaeAsteralesAsteraceae

(J.Anthony) Yu.J. Wang & L.M. Heng, comb. et stat. nov.

urn:lsid:ipni.org:names:77192776-1

≡CrepisgilliiS. Moorevar.hirsuta J. Anthony in Notes Royal Bot. Gard. Edinb. 18: 193. 1934 (Syntype: E00383690); ≡Soroserisgillii(S. Moore)Stebbinssubsp.hirsuta (J. Anthony) Stebbins in Mem. Torrey Bot. Club 19 (3): 44. 1940 (Syntype: E00383690); S. Y. Hu in Quart. Journ. Taiwan Mus. 21 (3–4): 166. 1968; ≡Soroserishirsuta (J. Anthony) C. Shih in Act. Phytotax. Sin 31: 446.1993; Flora Reipublicae Popularis Sinicae. 80 (1): 201. 1997.

#### Soroseris hookeriana subsp. occidentalis

Taxon classificationPlantaeAsteralesAsteraceae

(Stebbins) Yu.J. Wang & L.M. Heng comb. nov.

urn:lsid:ipni.org:names:60477691-2

≡Soroserisgilliisubsp.occidentalis Stebbins in Mem. Torrey Bot. Club. 19 (3): 44. 1940 (Type: K000250154); Babcock in Univ. Calif. Publ. Bot. 22: 922. 1937; S. Y. Hu in Quart. Journ. Taiwan Mus. 21 (3–4): 166. 1968; ≡Soroserisoccidentalis (Stebbins) Tzvelev in Bot. Zhurn. 92: 1753. 2007.

#### Soroseris hookeriana subsp. handelii

Taxon classificationPlantaeAsteralesAsteraceae

(Stebbins) Yu.J. Wang & L.M. Heng comb. nov.

urn:lsid:ipni.org:names:77192779-1

≡Soroserisgilliisubsp.handelii Stebbins in Mem. Torrey Bot. Club. 19 (3): 42. 1940 (Isotype: E00383689); S. Y. Hu in Quart. Journ. Taiwan Mus. 21 (3–4): 166. 1968.

## Supplementary Material

XML Treatment for Soroseris hookeriana subsp. gillii

XML Treatment for Soroseris hookeriana subsp. hirsuta

XML Treatment for Soroseris hookeriana subsp. occidentalis

XML Treatment for Soroseris hookeriana subsp. handelii

## Supplementary materials

Suppl. material 1 The main morphological difference amongst members of the *Soroserishookeriana* complex and the closely related species Data type: measurement This is an open access article distributed under the terms of the Creative Commons Attribution License (CC BY 4.0), which permits unrestricted use, distribution, and reproduction in any medium, provided the original author and source are credited.La-Mei Heng, Yu-Lin Zheng, Yong-Bao Zhao, Yu-Jin Wang

Suppl. material 2 The 50% majority rule consensus tree derived from Bayesian inference of the combined sequences of nuclear internal transcribed spacer, psbA-trnH and matK Data type: molecular data Explanation note: Posterior probabilities and bootstrap percentages are indicated above and below the branches, respectively. The samples named according to FOC (2011) or NCBI, Stebbins (1940) and the present study are listed from left to right. This is an open access article distributed under the terms of the Creative Commons Attribution License (CC BY 4.0), which permits unrestricted use, distribution, and reproduction in any medium, provided the original author and source are credited.La-Mei Heng, Yu-Lin Zheng, Yong-Bao Zhao, Yu-Jin Wang
