# Supplementary figures and images for: Radiation of members of the Soroserishookeriana complex (Asteraceae) on the Qinghai-Tibetan Plateau and their proposed taxonomic treatment
Source: PhytoKeys. 2018 Dec 20;(114):11–25. doi: 10.3897/phytokeys.114.29914 (PMC6308221; doi:10.3897/phytokeys.114.29914)

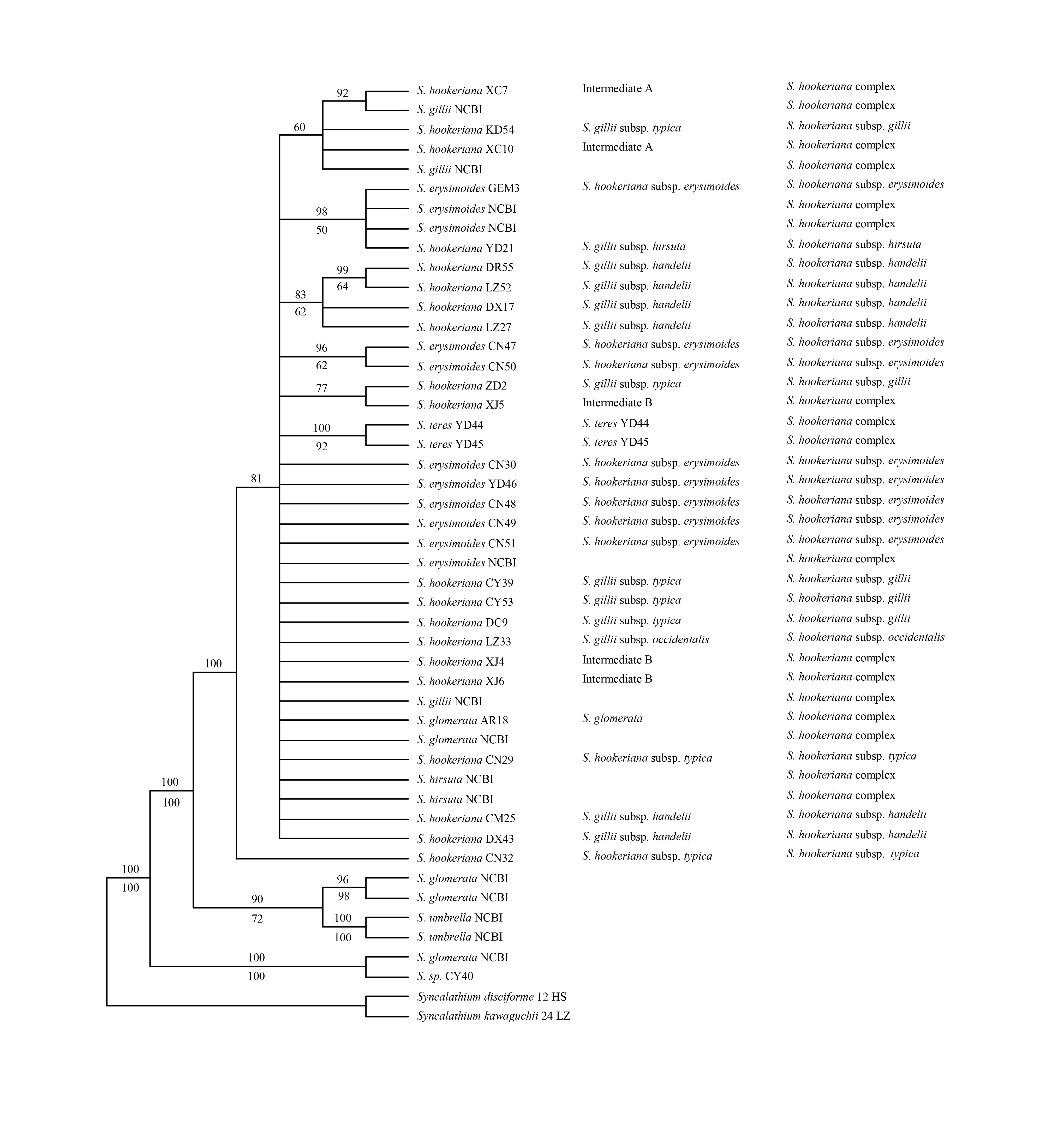

Supplement: Suppl. material 2 — The 50% majority rule consensus tree derived from Bayesian inference of the combined sequences of nuclear internal transcribed spacer, psbA-trnH and matK [file phytokeys-114-011-s002.jpg]
